# Propolis polyphenols: A review on the composition and anti-obesity mechanism of different types of propolis polyphenols

**DOI:** 10.3389/fnut.2023.1066789

**Published:** 2023-03-31

**Authors:** Liuying Zhu, Jinwu Zhang, Hui Yang, Guangyan Li, Hongyan Li, Zeyuan Deng, Bing Zhang

**Affiliations:** ^1^State Key Laboratory of Food Science and Technology, Nanchang University, Nanchang, China; ^2^Nanchang Concentric Purple Nest Biological Engineering Co., Ltd., Nanchang, China

**Keywords:** propolis polyphenols, plant origin, chemical composition, anti-obesity, mechanism

## Abstract

Obesity, one of the most common nutritional diseases worldwide, can lead to dyslipidemia, high blood sugar, high blood pressure, and inflammation. Some drugs have been developed to ameliorate obesity. However, these drugs may cause serious side effects. Therefore, there is an urgent need for alternative “natural” remedies including propolis. Studies have found that propolis has excellent anti-obesity activity in *in vitro* and *in vivo* models during the past decades, of which polyphenols are the key component in regulating weight loss. This review focused on the different polyphenol compositions of propolis from different regions and plants, the evidence for the anti-obesity effects of different types of propolis and its derivatives, discussed the impact of propolis polyphenols on obesity related signal pathways, and proposed the molecular mechanism of how propolis polyphenols affect these signal pathways. For example, propolis and its derivatives regulate lipid metabolism related proteins, such as PPARα, PPARγ, SREBP-1&2, and HMG CoA etc., destroy the formation of CREB/CRTC2 transcription complex, activate Nrf2 pathway or inhibit protein kinase IKK ε/TBK1, thereby affecting fat production and lipid metabolism; The effects of propolis on adipokines (adiponectin, leptin and inflammatory factors) were discussed. Additionally, the mechanism of polyphenols in propolis promoting the browning of adipose tissues and the relationship between intestinal microorganisms was summarized. These information may be of value to better understand how specific propolis polyphenols interact with specific signaling pathways and help guide the development of new drugs to combat obesity and related metabolic diseases.

## 1. Introduction

Obesity is a chronic metabolic endocrine disease regulated by dietary, genetic, behavioral, and environmental factors involved in unbalanced eating pattern and energy metabolism that result in increased body fat accumulation (1). Nowadays, people are living in life style, where obesity is spreading like an “epidemic” around the world. Moreover, obesity tends to be younger and has long been a global public health crisis (2). As early as 2012, World Health Organization warned that overweight and obesity have become the fifth leading cause of death globally. Obesity itself is not fatal, but it can cause various non-communicable complications, such as hyperlipidemia, coronary heart disease, hypertension, osteoarthritis, type II diabetes, and cancer (3–7).

The underlying cause of obesity is an imbalance in the relationship between energy intake and reward-punishment mechanisms, which induce obesity (8). At present, lifestyle interventions and drugs are commonly used in the treatment of obesity. Lifestyle changes can reduce weight in the initial stage, but long-term adherence is a huge challenge. Physiological and environmental factors can cause weight regain (up to 90% weight regain) (9). Medication can be effective for weight loss, but long-term use can cause a range of side effects, such as gastrointestinal discomfort, insomnia, dizziness, and constipation (10). To date, drug-based treatments for obesity and related diseases have remained limited, either producing harmful side effects or being inefficient. Therefore, there is an urgent need for alternative “natural” remedies.

Propolis is a natural resinous complex mixture produced by honeybees that contain bioactive compounds (11). It is a traditional folk medicine used in the treatment of various diseases and has biological activities such as antibacterial, antiviral, anti-inflammatory, local anesthetic, antioxidant, immunostimulant, anti-caries and anti-cancer (11, 12). Various biological and pharmacological effects of propolis activity have been reported to be related to phenolic compounds, and propolis polyphenols have been studied for weight loss over the past few decades (13, 14). Numerous studies have reported the weight loss effects of polyphenols in propolis in animals and humans (14, 15). This article reviews the polyphenol composition of different sources of propolis, and the evidence for their anti-obesity effects, and discusses their molecular mechanisms. It is hoped that this article will improve our understanding of the weight loss effects of propolis and facilitate future research.

## 2. Differences in phenolic composition of different propolis types

Propolis consists of 10% volatile substances, 50%–55% resin (mainly including flavonoids, phenolic acids and esters), 30%–40% Beeswax, 5%–10% pollen and other substances (16). The chemical composition of propolis is determined by the gum source plants and is affected by season, climate, bee species, collection methods, etc. (17, 18). At present, more than 300 compounds have been identified in propolis (17). According to the different source plants of gum, it can be divided into poplar type propolis, *Baccharis* type propolis, *Betula* type propolis, *Macaranga* type propolis, and *Dalbergia* type propolis, etc. At present, the research on the anti-obesity effect of propolis mainly focuses on three types of propolis, including poplar type propolis, *Baccharis* type propolis, *Dalbergia* type propolis. The following is a description of the three main botanical sources and representative components of propolis.

### 2.1. Poplar-type propolis

Poplar-type propolis is the widely used and studied type of propolis, commonly distributed in Europe, North America, most of Asia and parts of North Africa (19, 20). Propolis varies in compositions, with more than 300 compounds identified as (21): (a) free phenolic acids, such as caffeic acid, p-coumaric acid and ferulic acids; (b) esters of these acids, such as caffeic acid phenethyl ester (CAPE); (c) flavonoids (flavones and flavonols) such as crysin, luteolin, apigenin, and kaempferol (21).

**Figure 1:**
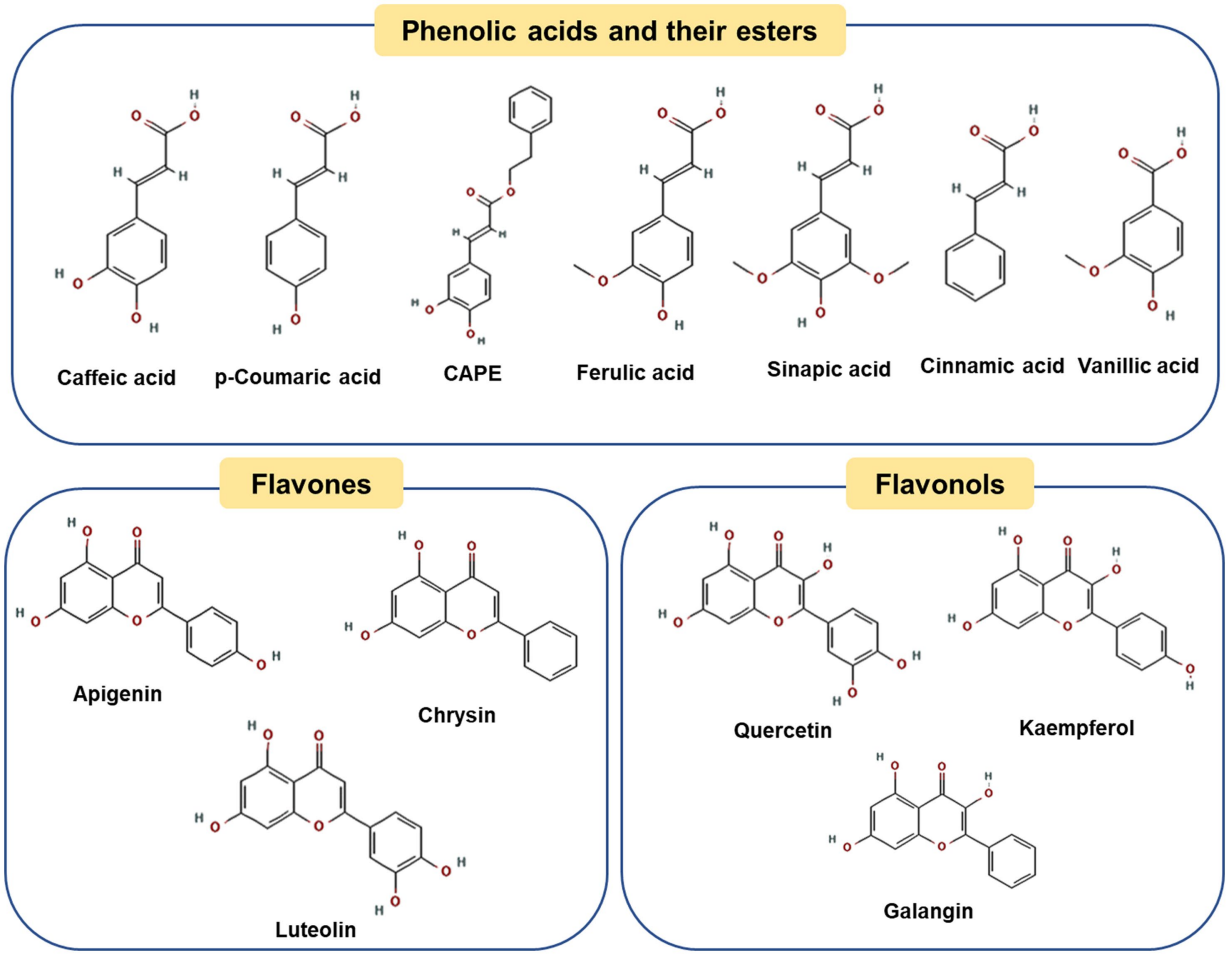
Chemical structures of the representative compounds in poplar-type propolis.

Figure 1 shows representative polyphenol compounds in poplar propolis. Caffeic acid and p-Coumaric acid are the main phenolic acids in water extracts of poplar propolis, with the contents reaching 76.9 ± 0.6 and 61.4 ± 0.3 mg/g, respectively (22). It is reported that caffeic acid can prevent diet induced hyperlipidemia and obesity in mice by regulating the expression of intestinal microorganisms and liver lipogenic genes (23, 24). Besides, the study found p-coumaric acid can be used against obesity by promoting thermogenesis of brown adipose tissue (25). The esters of caffeic acid, CAPE is one of the most characteristic polyphenols in ethanolic extracts of poplar propolis (about 11.2 ± 0.2 mg/g) (22). CAPE is a promising propolis ingredient. Several studies have shown that CAPE has many beneficial biological properties, including antioxidation (22), improving radiation protection (26), insulin resistance (27), and anti-obesity (28). Cardinault et al. explored the differential effects of three types of propolis alcohol extracts (Poplar, *Baccharis* and *Dalbergia*) on obesity in high-fat-fed mice, and found that only poplar propolis extract exerted a significant anti-obesity effect, suggesting that this difference may be related to its unique polyphenol compound (such as CAPE) (29). Moreover, *in vivo* and *in vitro* studies have found that CAPE can inhibit the differentiation of mouse preadipocytes and prevent adipogenesis (30, 31), and its specific mechanism will be discussed in the next chapter. In addition to phenolic acids, flavonoids, such as chrysin, galangin, apigenin and quercetin, exhibit excellent anti-obesity effects through different mechanisms (32–35).

### 2.2. Baccharis type propolis

Baccharis type propolis, mainly produced in Minas Gerais, Sao Paulo, Rio de Janeiro and Paraná in southeastern Brazil, is also known as “green propolis” due to its color ranging from greenish yellow to dark green (14). *Baccharis dracunculifolia* has been shown to be the most important botanical source of propolis (36, 37). Therefore, its chemical composition is very different from that of poplar-type propolis.

**Figure 2:**
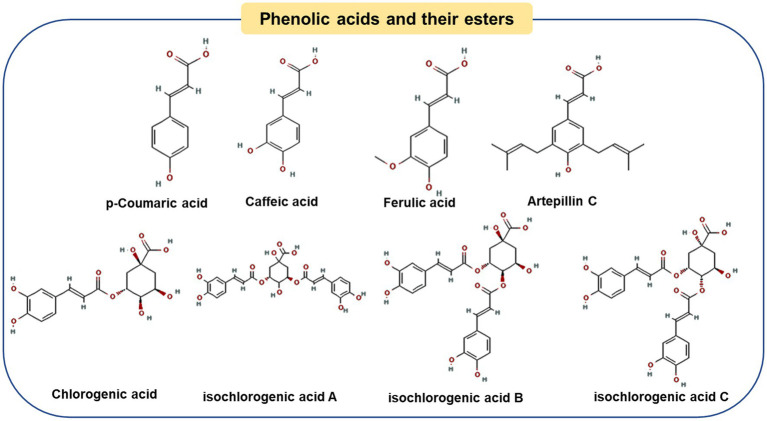
Chemical structures of the representative compounds in Brazilian green propolis.

Figure 2 shows representative components of *Baccharis* type propolis. Compared with poplar-type propolis, Brazilian green propolis was also rich in Artepillin C (Art-C) and chlorogenic acid, with contents of 91.84 ± 1.16 mg/g and 19.35 ± 0.37 mg/g, respectively (38). As one of the main chemical markers of green propolis, Art-C can regulate a variety of signaling pathways to prevent the development of chronic diseases, including anti-obesity (39), anti-inflammatory (40), gastroprotective (41) and immunomodulatory (42), etc. Studies have found that Art-C plays an anti-obesity role by regulating several pathways, including affecting brown adipocyte production and promoting white adipose tissue thermogenesis (discussed in the next chapter) (39, 43). Another phenolic acid, chlorogenic acid, has also been proved to improve obesity by preventing energy balance transfer and improving lipid metabolism in obese mice fed by high-fat diet (44, 45).

### 2.3. *Dalbergia* type propolis

**Figure 3:**
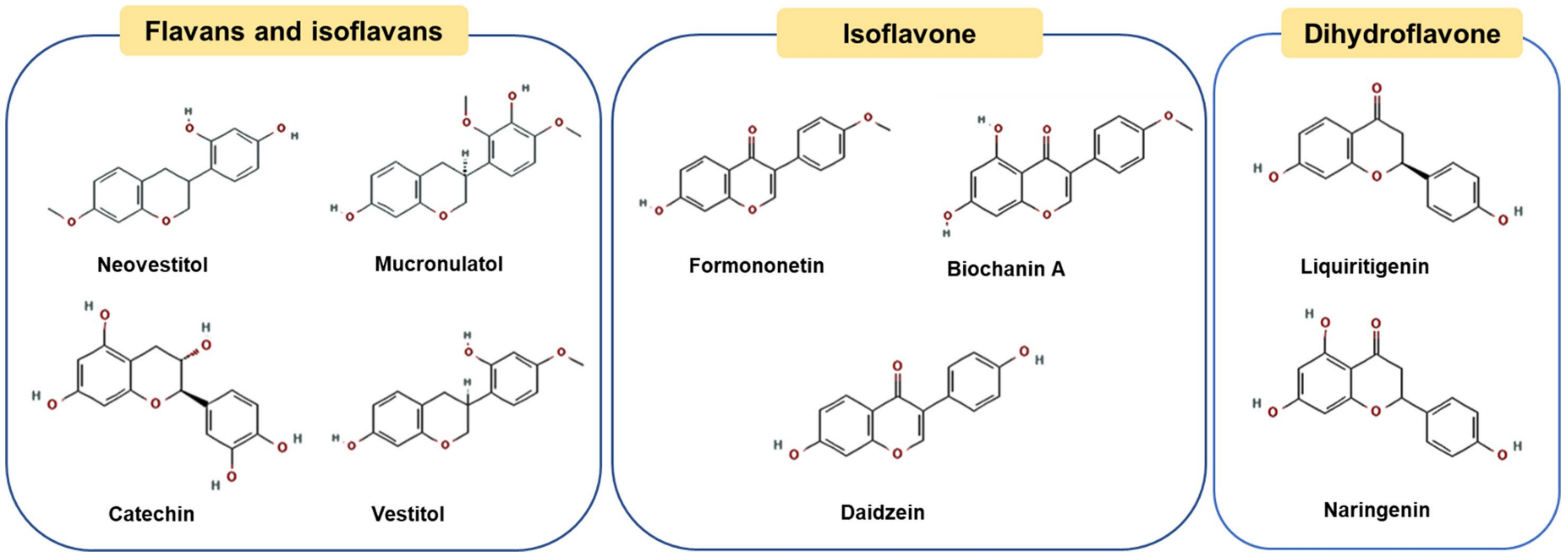
Chemical structures of the representative compounds in Brazilian red propolis.

*Dalbergia* propolis is mainly produced in Brazil, Cuba, Venezuela in South America and Mexico in North America (46–48). The main plant source has been determined as *Dalbergia ecastaphyllum* (L.) Taubert (49). This type of plant produces a red resin, which is collected by bees and used to produce propolis. Therefore, the color of *Dalbergia* propolis is often bright red and highly recognizable, also known as red propolis (50). Figure 3 shows the chemical structures of the representative phenolic compounds found in ethanol extract of red propolis. The main components of *Dalbergia* propolis are flavonoids (including flavanids, isoflavones and dihydroflavonoids) (51, 52). Isoflavones are the most abundant characteristic compounds of *Dalbergia* propolis different from poplar type and green propolis, Bueno-Silva et al. indicated that formononetin is the most abundant compound in Brazilian red propolis and resin, at 112.78 ± 9.07 μg/g and 77.4 ± 1.05 μg/g, respectively (53). They play a role in different physiological processes and play a variety of functions, including anti-obesity (54), anti-bacterial (55), anti-inflammatory and anti-cancer effects (52). Marcelle F. Prata and co-workers explored the effect of extract of Brazilian red propolis (mainly contained isoflavones) on hypolipidemic and anti-obesity in dyslipidemia mice model, and found that the extract of Brazilian red propolis had a hypolipidemic effect on rodent models with dyslipidemia, and minimized the effects of high-fat diet on body weight parameters and abdominal fat accumulation in mice (56). In addition, *in vitro* experiments showed that the ethanolic extracts of Brazilian red propolis could affect the differentiation of 3T3-L1 preadipocyte cells and the expression of adipokine (54). Although there are few studies, it can be found that red propolis extract has a promising future as a dietary supplement for the prevention and treatment of obesity and obesity-related diseases.

## 3. Mechanisms of anti-obesity by propolis polyphenols

Currently, most studies on propolis against obesity used cellular or animal models, but there are very few clinical studies on the subject. This section identified 26 preclinical and 2 clinical studies, focusing on the effects of propolis and its extracts on obesity and summarized their anti-obesity mechanisms (Table 1).

**Table 1:** Preclinical and clinical studies investigating the effect of propolis in anti-obesity.

Administrated material	*In vitro*/*in vivo* model	Treatment/Dose	Effect	Anti-obesity mechanism	Reference
Brazilian green propolis	HFD-induced obese Wistar rats	a high-fat diet supplemented with 0.5% (w/w) and 0.05% (w/w) propolis	↓ Fat accumulation; ↓ PPARγ; ↑ lipid metabolism-related proteins, (PPARα, SREBP-1 and HMG-CoA)	Affects fat accumulation and lipid metabolism	(57)
	HFD-induced C57BL/6 mice	2% propolis for 14 weeks	↓ Body weight; ↓ epididymal fat mass, ↓ subcutaneous fat mass; ↑ fecal weight; ↑ fecal fat content		(58)
Ethanolic extracts of Brazilian green propolis	HFD-induced obese C57BL/6N mice	5 mg/kg or 50 mg/kg twice daily for 10 days	↓ Body weight gain; ↓ visceral adipose weight; ↓ mRNA expression associated with fatty acid biosynthesis (SREBP-1& SREBP-2; ACAC&FAS)		(59)
	C57BL/6JHamSlc-*ob/ob* mice	100 mg/kg, ip, twice a week for 12 weeks	↓ Mesenteric adipose tissue mass, ↓ plasma cholesterol		(60)
Ethanolic extracts of Croatia propolis	HFD-induced C57BL/6 mice	50 mg/kg ethanol extract of propolis for 30 days	↓ Body weight; ↓ hepatic lipid accumulation; ↓ plasma atherogenic index		(61)
Chinese propolis	HFD-induced C57BL/6 mice	150 and 300 mg/kg propolis for 9 weeks	↓ Body weight; ↑ insulin resistance. ↓ triglyceride accumulation		(62)
Art-C	HEK293T cells	0–0.2% Art-C for 6 h	↓ CRTC2 interact with CREB; ↓ acetyltransferase activity of ↓ CBP; gluconeogenesis		(63)
	HFD-induced C57BL/6 mice and C57BL/KsJ-*db/db* mice	intraperitoneal injection of synthesized Art-C (10 or 20 mg/kg) for 5 weeks	↓ Body weights; ↓ hyperlipidemia; ↓ SREBP-mediated lipid synthesis; ↑ insulin resistance		
CAPE	HFD-induced C57BL/6 mice	0.02, 0.1, or 0.5% CAPE (w/w) for 5 weeks	↓ Body weight; ↓ epididymal fat mass; ↓ adipogenesis		(28)
	3T3-L1 preadipocytes	(10, 20, 40 μM) of CAPE for 8 days			
Ethanolic extracts of Brazilian green propolis	Differentiated 3T3-L1 adipocytes	100 μg/ml of the propolis extract for 4 h	↑ Leptin expression	Affect the expression of adipocytokine and inhibit adipogenesis	(64)
	HFD-induced obese C57BL/6 mice	intraperitoneal injection 100 mg/kg twice a week for 5 weeks	↓ Feeding; ↑ leptin mRNA		
CAPE	3T3-L1 mouse fibroblast cells	(10, 25, 50 mM) of CAPE for 7 days	↓ Leptin; ↓ resistin; ↓ TNF-alpha		(31)
		(10, 25, 50 mM) of CAPE for 7 days	↓ PPARγ; ↓ C/EBP-α, ↓ Fas & aP2		(30)
	ASCs-derived adipocytes	Administration of 10 μM CAPE every 3 days	↑ Insulin sensitivity; ↑ adiponectin; ↓ proinflammatory cytokine mRNA level (TNF-α, IL-1β, IL-6, IL-8)		(65)
Trigona propolis	Central obesity human (*n* = 10)	60 mg/days propolis and divided into 3 capsules	↓ Leptin level		(66)
Ethanolic extracts of Brazilian red propolis (EERP)	3T3-L1 preadipocytes	20 μg/ml EERP for 3 days	↑ PPAR γ transcriptional activity; ↑ adiponectin promoter activity; ↓ differentiation; ↑ adiponectin production		(54)
Various Brazilian propolis-derived components (Art-C, acrylic acid, p-coummaric acid drupanin, baccharin)		25 μM of each Brazilian propolis-derived components for 15 h	↑ CAdiponectin		(67)
Art-C		10 μM of Art-C for 9 days	↑ Adipocyte differentiation; ↑ adiponectin expression		(68)
Ethanolic extracts of Brazilian red propolis	3T3-L1 preadipocytes	ethanolic extracts of red propolis (0–100 mg/ml) for 24 h	↑ Adiponectin mRNA		(54)
Poplar propolis ethanolic extract (PPEE)	HFD-induced C57BL/6J mice	20 mg PPEE per mouse per day for 12 weeks	↓ Adipocyte hypertrophy, ↓ body weight gain; ↑ glucose homeostasis; ↑ promoting fatty acid oxidation genns (Ppara, Cpt1a, Atgl, Mcad and Lcad); white ↑ adipose tissue browning related genes (Ucp1, Ppargc1a Prdm16 and Cidea); ↓ inflammation markers (Tnfa, Ccl5 and Ccl2)		(69)
	3T3-L1 preadipocytes	3–30 μg/ml PPEE for 24 h	↑ Nrf2 Pathway (Nrf2, Gclc, Gclm, and Nqo1); ↑ a Nrf2 response element (ARE)		
Chrysin	HFD-induced C57BL/6 mice	60, 100, and 200 mg/kg Chrysin for 30 days	↓ IKKε/TBK1; ↓ body weight; ↓ insulin resistance, ↓ key lipogenic gene (Fasn1 and SCD-1); ↓ inflammatory gene (Tnfa and CXCL2)		(70)
Ethanolic-extracted poplar propolis	54 eligible male participants	Take 450 mg ethanol extracted propolis twice a day before lunch and dinner for 4 weeks	↓ Inflammation status (IL-6 and IL-10); ↓ oxidative stress index		(71)
Art-C isolated from Brazilian propolis	Obese C57BL/6J mice	10 mg/kg body weight for 28 days	↑ Thermogenesis of beige adipocytes; ↑ creatine metabolism pathways	Induction of Brown/Beige Adipocytes	(39)
	C57BL/6J mice	5 and 10 mg/kg propolis for 4 weeks	↑ Brown-like adipocytes; ↑ UCP1; ↑ PRDM16 proteins.		(43)
	C3H10T1/2 cells and primary inguinal WAT (iWAT)-derived adipocytes	(1, 5, 10 μM) of Art-C for 8 days			
Crude Brazilian propolis	HFD-induced C57BL/6 mice	0.2% crude propolis for 5 weeks	↑ Gut microbiota homeostasis; ↓ TLR4; ↓ circulating LPS; ↓ inflammatory response	Regulate the structure and metabolism of gut microbes	(72)
Ethanolic extracts of Chinese propolis (EEP)	HFD-induced C57BL/7 mice	1% or 2% EEP for 12 weeks	↓ Body weight; ↓ liver fat accumulation; ↓ proinflammatory cytokines and bacteria; ↓ insulin resistance; ↑ glucose tolerance; ↑ anti-obesity and anti-inflammatory bacteria		(73)
Chinese propolis	HFD-induced C57BL/8 mice	150 and 300 mg/kg propolis for 9 weeks	↓ Body weight; ↓ insulin resistance ↑ Gut microbiota homeostasis		(62)
Polyphenol mixture extracted from poplar-type propolis	*In vitro* fermentation (fecal material from different 5 donors)	–	↑ Gut microbiota homeostasis; ↑ SCFAs		(74)
Brazilian green propolis	non-diabetic heterozygous Db/m mice and Db/Db mice	0.08, 0.4, and 2% propolis in standard diet for 8 weeks	↓ Non-alcoholic fatty liver disease activity score; ↓ Genes related to inflammation and fatty acid metabolism; ↑ Butyricicoccus and Acetivibrio genera		(75)
CAPE	Fxr^fl/fl^ and intestine-specific Fxr-null (Fxr^ΔIE^) mice on a C57BL/6 genetic background	75 mg/kg/days CAPE for 8 weeks	↓ BSH-producing bacteria; ↓ BSH activity; ↑ T-β-MCA; ↓ intestinal FXR signaling; ↑ GLP-1; ↓ ceramide synthesis; ↓ lipid synthesis		(76)

### 3.1. Affects adipogenesis, fat accumulation and lipid metabolism

**Figure 4:**
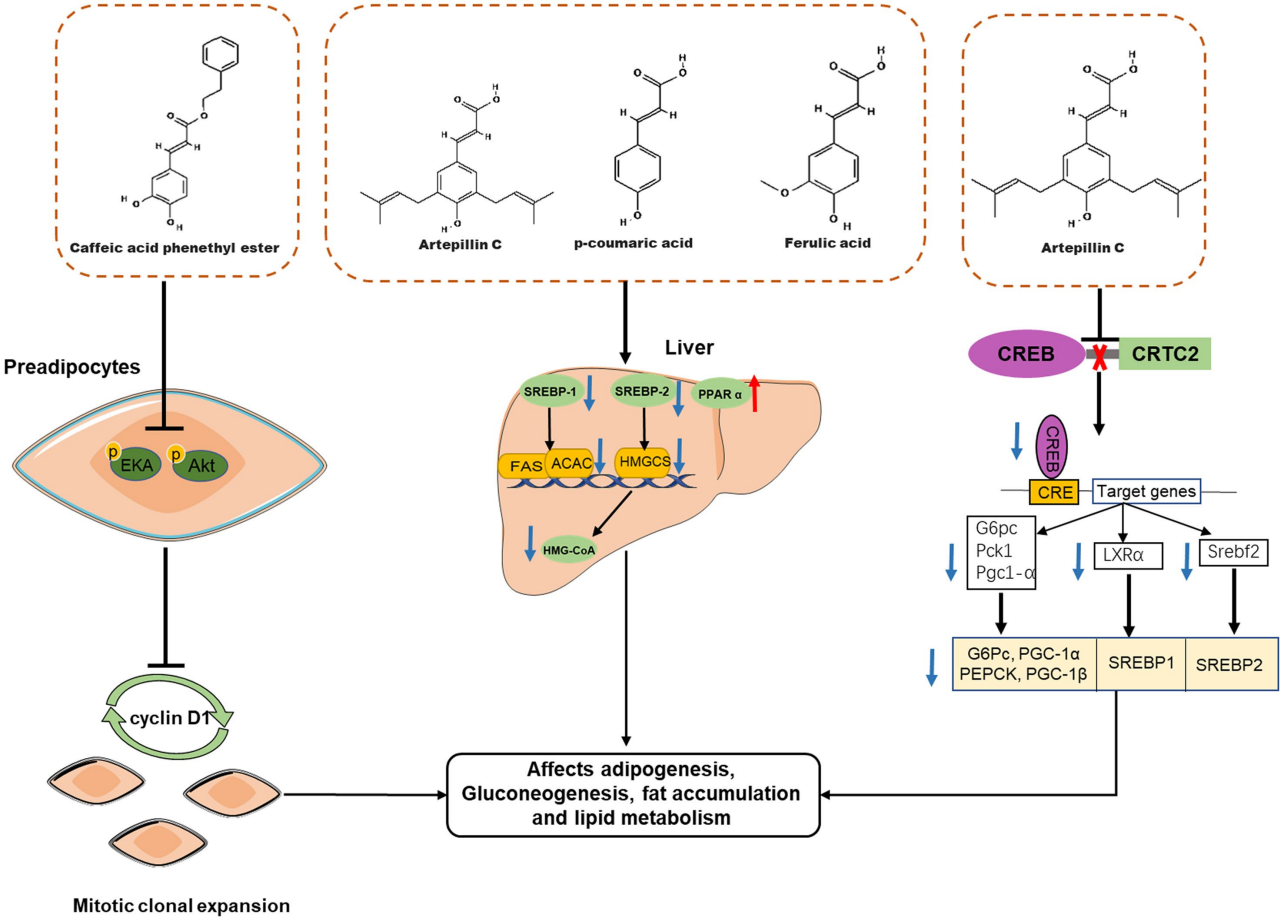
Effects of propolis-derived compounds on adipogenesis, fat accumulation and lipid metabolism. Caffeic acid phenethyl ester (CAPE) represses clonal expansion by disturbing the ERK/Akt-cyclin D1 cascade. Propolis may improve body fat accumulation and dyslipidemia by affecting the expression of fat accumulation and lipid metabolism-related proteins, such as peroxisome proliferator-activated receptor α (PPAR α) Sterol-regulatory element binding protein-1(SREBP-1), SREBP-2, and 3-Hydroxy-3-methylglutaryl-coenzyme A (HMG-CoA). Artepillin C exert anti-obesity effects by suppressing CREB/CRTC2-mediated both gluconeogenic and SREBP transcriptions.

So far, numerous *in vitro* and *in vivo* studies have demonstrated that propolis affects body weight and adipose tissue weight in obese animals by affecting adipogenesis, fat accumulation and lipid metabolism (Figure 4). Ichi et al. reported that feeding pellets containing 0.5% (w/w) Brazilian propolis for 8 weeks reduced fat accumulation and serum cholesterol and triglyceride levels in high-fat-fed rats (57). In addition, propolis intake also affected lipid metabolism-related proteins in mice, such as peroxisome proliferator-activated receptor alpha (PPAR α), sterol-regulatory element binding protein 1 (SREBP-1), and 3-Hydroxy-3-Methylglutaryl-Coenzyme A (HMG-CoA), therefore, propolis may improve body fat accumulation and dyslipidemia by affecting the expression of fat accumulation and lipid metabolism-related proteins (57), which was the same result as another report (59). Therapeutic effects of Brazilian propolis extract on anti-obesity and dyslipidemia have also been observed in a single-gene mutant obesity model (60). Besides Brazilian propolis, propolis from other geographical areas also has weight loss effects. For example, Chinese propolis reduced the body weight of high-fat diet mice in a dose-dependent manner and reversed the liver weight loss and triglyceride accumulation associated with liver steatosis (62); Oral administration of ethanolic extract of Croatian propolis (50 mg/kg, for 30 days) was also shown to reduce body weight and hepatic lipid accumulation in high-fat diet C57BL\6N mice, and improve mouse plasma atherogenic index (61). However, although the intervention of propolis is related to the increase of lipid metabolism related proteins, as a mixture, the details of how specific components of propolis affect the lipid metabolism of the body are still unknown. Consequently, the anti-obesity effect of propolis-derived chemicals (such as CAEP) was investigated. Several studies have found that CAPE inhibits adipocyte differentiation. For example, CAPE can significantly inhibit the expression of proteins related to lipid metabolism in adipocytes, such as PPAR-γ, adipocyte-specific fatty acid binding protein (aP2), C/EBP-α and fatty acid synthase, thereby reducing the deposition of triglycerides in 3T3-L1 cells after MDI stimulation (30, 31). The molecular mechanism by which CAPE inhibits adipocyte differentiation has also been investigated. Shin et al. found that 40 μM CAPE delayed the cell cycle progression of MDI-stimulated 3T3-L1 cells, thereby inhibiting mitotic clonal expansion (28). At the same time, CAPE also blocked the phosphorylation of ERK and Akt in 3T3-L1 cells, resulting in the inhibition of the expression of cyclin D1 downstream (31, 77). Therefore, CAPE can inhibit fat production by interfering with the early phase of fat production.

Although some characteristic compounds in propolis have been reported to ameliorate metabolic syndrome, it remains a serious challenge to identify new targets of propolis active compounds and explore their underlying mechanisms. Recently, a new molecular target of propolis to improve metabolic syndrome has been found, that is, Art-C protects mice from obesity induced by high-fat diet, enhances insulin sensitivity and improves glucose and lipid metabolism by inhibiting CREB/CRTC2-mediated both gluconeogenic and SREBP transcription (63). Excitedly, Art-C has been designed and modified to form a new compound A57, which shows higher inhibitory activity on CREB-CRTC2 association and better ability to improve insulin sensitivity in obese animals (63).

### 3.2. Affect the expression of adipocytokine

**Figure 5:**
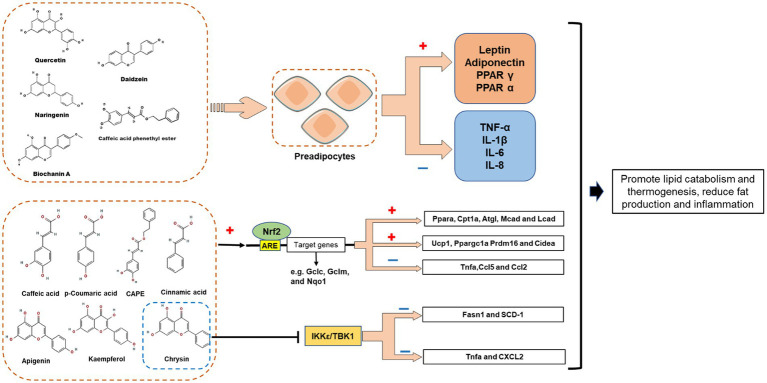
Proposed mechanisms demonstrating the role of propolis-derived compounds in the regulation of adipocytes lipid metabolism *via* induction of PPARs, adiponectin and leptin. Restore the function of mast adipocytes by reducing inflammatory cytokines. Propolis polyphenols may promote the expression of fatty acid oxidation gene (Ppara, Cpt1a, Atgl, Mcad, and Lcad) and white adipose tissue browning gene (Ucp1, Ppargc1a Prdm16 and Cidea) by activating Nrf2 pathway, while reducing the expression of inflammation gene (Tnfa, Ccl5 and Ccl2); Chrysin may inhibit I-kappa-B kinase epsilon (IKK ε) and TANK-binding kinase 1 (TBK1) to reduce the expression of key adipogenesis genes and inflammation genes, thereby reducing adipogenesis and inflammation.

For a long time, it was thought that the fat is simply a place to store fat. Recent studies have shown that adipocytes have a secretory function and can secrete various physiologic active substances, which are collectively known as adipocytokine. At present, the recognized adipocytokine can be divided into two categories: one is specifically expressed in adipose tissue, such as leptin and adiponectin; The other is non-specific expression of adipose tissue, such as tumor necrosis factor (TNF α) and interleukin-6 (IL-6). Research has shown that propolis and its extract can play an anti-obesity role by influencing the secretion of adipocytokine (Figure 5).

Leptin acts on the weight regulation center of the hypothalamus, leading to decreased appetite and increased energy consumption, thereby reducing fat deposits and inhibiting body weight gain (78). Kohei et al. studied the effect of Brazilian green propolis ethanol extract (EEGP) on leptin expression *in vivo* and *in vitro*, and found that EEGP (100 μg/ml) significantly increased the leptin expression of 3T3-L1 adipocytes (64). Similarly, intraperitoneal injection of EEGP (100 mg/kg, twice a week for 5 weeks) strongly inhibited the feeding of C57BL/6 mice, and tripled the leptin expression in epididymal adipose tissue (64). However, another report describes the effect of CAPE on leptin expression in 3T3-L1 adipocytes (31). CAPE inhibited leptin expression in 3T3-L1 cells in a dose-dependent manner (31), and accompanied by the down-regulation of insulin receptor substrate-1 (IRS-1) (31), which is a prerequisite for adipocyte differentiation (79). Hence, the CAPE-induced leptin reduction in 3T3-L1 cells appears to be due to insufficient cell differentiation. Actually, obesity also has a persistent increase in leptin that produces leptin resistance, affects glucose intolerance, and will become a determinant of diabetes, so for this type of obesity, controlling leptin as an intervention target may have the opportunity to prevent various chronic diseases (66). In a quasi-experimental study, subjects with central obesity and normal weight were collected to determine whether honey and propolis can decrease leptin levels in patients with central obesity (15). The study found that honey and propolis can reduce leptin levels in participants with central obesity, indicating that these bee products may become dietary supplements for patients with central obesity (15).

Besides, propolis and propolis-derived chemicals also modulate the expression of other adipokines (Table 1). Adiponectin is a beneficial adipokine that can regulate energy homeostasis, glucose metabolism, and fat metabolism in organisms (80). It was found that ethanolic extract of Brazilian red propolis (EERP, 20 μg/ml for 3 days) may activate the adiponectin promoter through PPARγ, thereby promoting the expression of adiponectin mRNA in post-confluent 3T3-L1 preadipocytes (54). The same report also demonstrated that EERP also reversed the inhibiting effect of TNF-α on adiponectin expression in differentiated 3T3-L1 cells (54). Other studies have also reported the up-regulation of adiponectin in adipocytes by other propolis derivatives. For example, Art-C (10 or 25 μM) significantly enhanced adiponectin expression (1.5–2.0-fold) in 3T3-L1 cells (67, 68). Furthermore, although CAPE (10 μM) decreased leptin expression, it more than doubled adiponectin expression in human ASC-derived adipocytes (65). Taken together, several polyphenolic compounds in propolis positively regulate adiponectin expression in adipocytes.

Apart from the beneficial adipokines, propolis can affect harmful adipokine expression. CAPE (10 μM) attenuated LPS-mediated effects and significantly down-regulated the expression of pro-inflammatory cytokines (TNF-α, IL-1β, IL-6, and IL-8) in ASC-derived adipocytes (65). In addition, in differentiated 3T3-L1 cells, CAPE had a significant inhibitory effect on TNF-α as well, although a higher dose (50 μM) was required (31). It was found in the same report that CAPE (25 or 50 μM) also decreased resistin mRNA and intracellular protein levels in 3T3-L1 cells (31). Recently, studies have shown that propolis polyphenols may play an anti-obesity role with the Nrf2 pathway, I-kappa-B kinase epsilon (IKK ε) and TANK binding kinase 1 (TBK1). Cardinault et al. studied the preventive effect of ethanol extract of poplar propolis (PPEE) on obesity and related metabolic diseases (69). The results showed that PPEE could prevent diet induced obesity, improve glucose homeostasis, promote lipid metabolism and thermogenesis, and significantly reduce the expression of inflammatory genes (such as Tnfa, Ccl5 and Ccl2), accompanied by the activation of Nrf2 pathway (69). Another study found that Chrysin can reduce hepatic IKK ε/TBK1 expression, promote triglyceride hydrolysis and oxidation, inhibit fat production and inflammation, indicating IKK ε/TBK1 may be one of the pathways of chrysin’s anti-inflammatory and insulin sensitivity (70). Nevertheless, the propolis and its derivatives reported in the above study have a regulatory effect on the two pathways of Nrf2 and IKK ε/TBK1, but the specific targets it acts on, and how to exert anti-obesity effects through specific targets in the pathway, are unknown. Moreover, a triple-blind randomized clinical trial involving 54 male military cadets reported that propolis intervention effectively reduced the oxidative stress and inflammation (IL-6, IL-10) of the subjects after vigorous activities, demonstrate that supplementation with propolis might have beneficial effects on oxidative stress and inflammation status following intense physical activities while not affecting athletic performance in healthy active subjects (71).

### 3.3. Induction of brown/beige adipocytes

**Figure 6:**
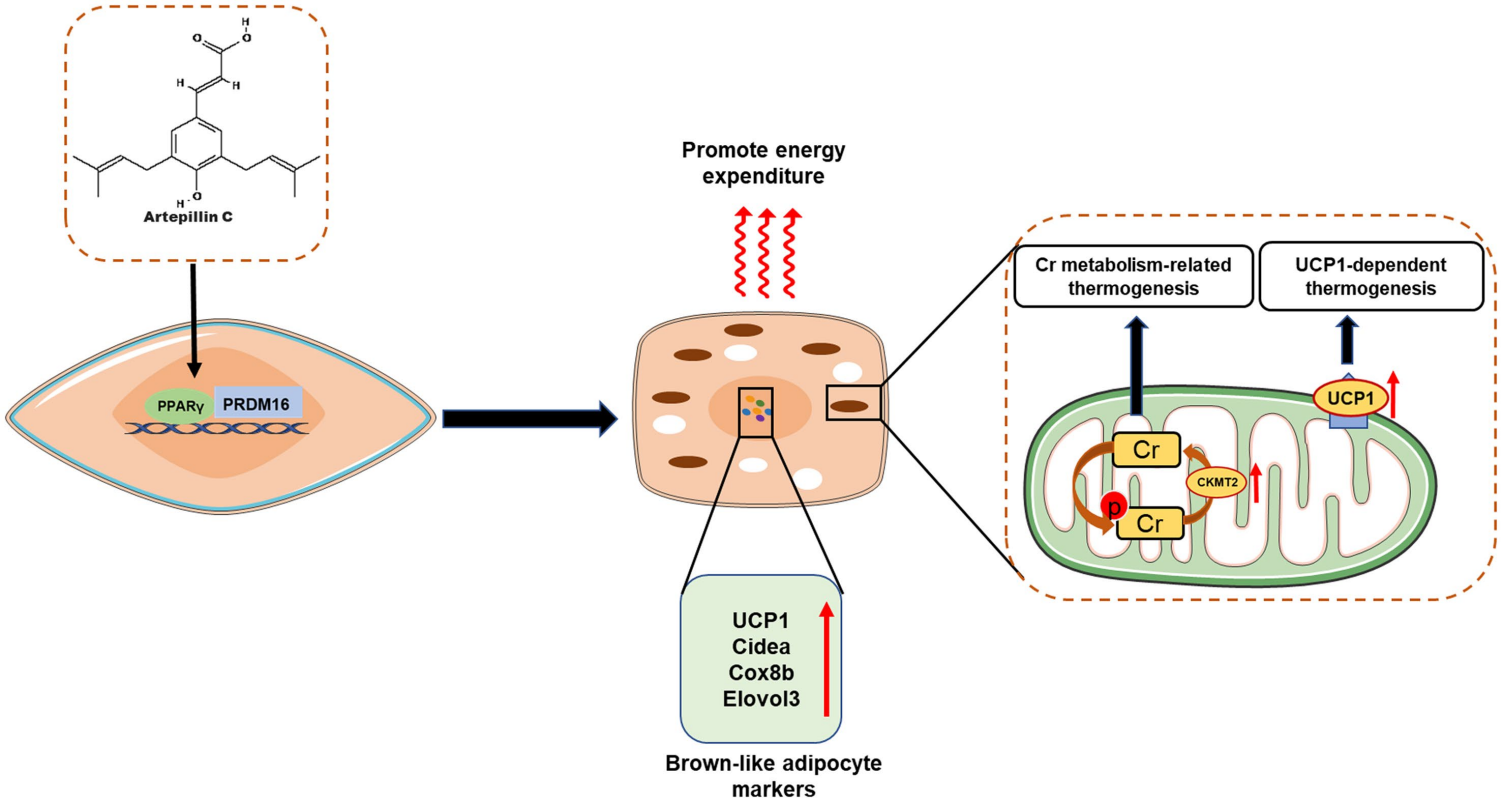
Artepillin C (Art C) induces brown-like adipocytes by activating peroxisome proliferator-activated receptor γ (PPAR γ) and stabilizing PRD1-BF-1-RIZ1 homologous domain-containing protein-16 (PRDM16); Art C significantly promotes the expression of genes related (UCP1, Cidea, Cox8b, and Elovl3) to brown adipocytes to induce browning of white adipose tissue, and promotes adipocyte thermogenesis by influencing creatine (Cr) metabolism–related and UCP1-dependent thermogenic pathway.

Mammals have two types of adipose tissue with distinct physiological functions, namely white adipose tissue (WAT) and brown adipose tissue (BAT): WAT stores excess energy in the form of ATP, while BAT is characterized by the expression of thermogenic uncoupling proteins 1 (UCP1) promotes calorie production to consume excess energy, thereby inhibiting weight gain and metabolic disease (81). Recent studies have shown that brown-like adipocytes, also known as beige or brite cells, are an inducible form of brown adipocytes present in white adipose tissue that share many biochemical and morphological features with brown adipocytes (82, 83). Since these cells are innately capable of releasing excess energy, a new strategy to induce brown/beige adipocytes in WAT may be one of the most feasible approaches to prevent and treat obesity and related diseases (84, 85). This has also brought attention to dietary factors or other diet-derived factors that may contribute to the induction of brown/beige adipocyte proliferation. Recent studies have found that Brazilian propolis-derived such as Art-C, can achieve anti-obesity effects by inducing browning and thermogenesis of white adipocytes (Figure 6).

Through *in vivo* and *in vitro* experiments, Nishikawa et al. investigated the effect and mechanism of Art-C induced browning of white adipocytes (43). *In vitro* experiments, Art-C (1–10 μM) significantly increased the mRNA level of brown adipocyte markers and the protein levels of UCP1 and PRDM16 in a dose-dependent manner (43). Further studies found that this significant induction was achieved by activating PPARγ and promoting the stabilization of PRDM16 protein (43). Similarly, in animal experiments, oral administration of Art-C (5 or 10 mg/kg for 4 weeks) can significantly induce brown like adipocytes in mouse inguinal adipose tissue, accompanied by significant expression of UCP1 and PRDM16 proteins, which further verifies the results of *in vitro* experiments (43). Recently, the same group also found that co-administration of Art-C (5 mg/kg) and curcumin (1.5 mg/kg) synergistically promoted the induction of brown adipocytes in inguinal adipose tissue, and believed that this synergistic effect was associated with norepinephrine produced by murine macrophages (86), but the specific molecular mechanism needs to be further explored. In addition, recent research of Nishikawa team also proposed that Art-C induced thermogenesis is associated with the thermogenesis pathway related to creatine metabolism (39). The research found that Art-C (10 mg/kg, 28 days) significantly induces the thermogenesis of beige adipocytes in inguinal adipose tissue. However, this induction effect is blocked by creatine metabolism inhibitors, indicating that Art-C may achieve energy consumption by significantly up regulating the expression of enzymes related to creatine metabolism in thermogenesis pathway (39). However, the relative contributions of UCP1-dependent and Cr-metabolism-related pathways to the observed Art-C-induced thermogenesis remain unclear. Further studies and mechanism investigations are needed to clarify the intrinsic relationship between Art-C and the two pathways, and UCP1-KO and WAT-specific GATM KO mice may be a powerful tool for subsequent studies (87).

### 3.4. Regulate the structure and metabolism of gut microbes

Gut microbiota are considered to be a metabolic “organ” involved in regulating energy balance, sugar and lipid metabolism (88, 89), and there is increasing evidence that microbiota regulation is associated with obesity (90). Polyphenolic compounds have been shown to have a very low absorption rate in the front end of the gut, only 5%–10%, and the rest of the unabsorbed polyphenols (90%–95%) reach the colon in high concentrations, where they are broken down by the gut microbiota and degraded into smaller phenolic compounds, which further exert a series of biological effects (91, 92). Therefore, polyphenolic compounds may exert biological effects such as regulating sugar and lipid metabolism, antioxidant and anti-inflammatory by regulating the composition and metabolism of gut microbiota (92–94). So, as the most abundant component in propolis, do propolis polyphenols exert anti-obesity effects by regulating intestinal microbes?

A few studies suggest that the anti-obesity effects of polyphenols in propolis may be related to changes in the gut microbiome (Table 1). Roquetto et al. investigated the effects of green propolis on gut microbiota composition and anti-inflammatory effects in mice fed a high-fat diet, and found that 0.2% crude propolis repaired high-fat diet-induced gut microbial disturbances and reduced circulating LPS levels and inflammatory response (72). Similarly, another study suggested that propolis may mediate anti-obesity effects by modulating gut microbiota composition and function (73). The study found that dietary supplementation with 1% or 2% ethanol extract of propolis reduced high-fat diet-induced weight gain and hepatic fat accumulation. Besides, it improved glucose tolerance and lipid profile, accompanied by an increase in anti-obesity and anti-inflammatory bacteria, such as *Intestinimonas* genera and *Parabacteroides distasonis* species and a decrease in pro-inflammatory bacteria, such as *Faecalibaculum* genera and *Bacteroides vulgatus* species (73). Recent study using Db/Db mice reported propolis can improve sarcopenic obesity by regulating lipid metabolism disorder and inflammation, regulate intestinal microecology, and increase the abundance of intestinal microbiota related to pentose phosphatase pathway and glycerol metabolism (75). Furthermore, a recent study first reported the therapeutic effect of Chinese propolis (CP) on obesity, and the results showed that dietary CP supplementation significantly improved obesity-related physiological indicators such as weight gain, insulin resistance, hepatic steatosis and triglyceride accumulation (62). Interestingly, the effect of CP on the microbiome structure and metabolism of mice varied by gender (62). It was found that, regardless of gender, CP significantly reduced the abundance of *Alistipes*, which is the main producer of LPS in mice, but only increased *Lactobacillus* and the level of propionic acid in male mice (62). In addition to the structure of intestinal microorganisms, the change of its metabolite short chain fatty acids (SCFA) is also very important for obesity (95). SCFA is absorbed in the intestine and plays a regulatory role in intestinal physiology, metabolism and immunity as a regulator of energy intake and inflammation (96). A recent study determined the effects of poplar propolis polyphenol mixtures on the composition and function of gut microbiota obtained from the stools of five different donors, including obese children (74). The results showed that propolis could significantly increase total gut microbial SCFA production in obese children, but did not result in a significant increase in propionic acid concentrations (74). Previous studies have shown that higher concentrations of propionic acid are associated with higher android-to-gynoid fat ratio, which is a risk factor for children’s metabolism and cardiovascular disease (97). Propolis polyphenol does not lead to propionic acid concentration, suggesting that propolis may play a protective role in obese subjects, or at least not worsen the situation (74). However, further studies are needed to understand how propolis polyphenols exert their anti-obesity effects by regulating the structure and metabolism of gut microbiota. A recent study has elucidated the therapeutic effect of CAPE on nonalcoholic fatty liver disease (NAFLD) through the regulation of gut microbes and its potential mechanism (76). It was found that oral administration CAPE inhibited bile salt hydrolase (BSH) activity by reducing the abundance of BSH-producing bacteria, such as *Parabacteroides* (76). The inhibition of CAPE in BSH lead to the increase of T-β-MCA, which inhibits intestinal FXR signal, reduces ceramide synthesis and promotes GLP-1 secretion. This pathway has been further verified in intestinal FXR-deficient mice (76). Thus, CAPE improves NAFLD by inhibiting bacterial BSH activity, altering bile acid composition and modulating the intestinal FXR signaling-ceramide axis. Nevertheless, although this study reveals the critical role of the gut microbiota during CAPE treatment, details about how CAPE specifically inhibits bacterial BSH activity and selectively modulates intestinal FXR signaling remain unknown. In addition, although intestinal FXR deficiency is associated with elevated serum GLP-1 levels, earlier studies have reported that TGR5 directly promotes GLP-1 secretion (98, 99), while FXR indirectly regulates GLP-1 secretion through mechanisms that rely on downstream TGR5 signaling. Therefore, more in-depth studies are needed to explore the mechanism of TGR5 in CAPE-mediated upregulation of Gcg and GLP-1.

## 4. Perspectives

Past studies have accumulated sufficient knowledge on the potential anti-obesity benefits of polyphenols in propolis, but there are still many pressing questions and challenges that need to be addressed.

First, the polyphenolic compounds in propolis, which play a major therapeutic role, are believed to have low toxicity and side effects (100). However, propolis is a complex mixture, albeit purified, so we cannot completely rule out the possibility that propolis could have adverse effects on patients. In fact, a human study has shown that propolis may be a skin allergen (101). To avoid the harmful effects of propolis, comprehensive monitoring of biological effects appears to be important. Unfortunately, there are few studies using omics approaches, such as transcriptomics, proteomics and metabolomics, to investigate the anti-obese effect of propolis polyphenols. According to these methods, researchers can more freely obtain information about the absorption and metabolism of propolis components in the body in experimental models, and to understand the information about the possible adverse effects of propolis components. In addition, omics data may also help to reveal the potential beneficial effects of propolis in obesity models, which may further elucidate the mechanism of action of effector molecules.

Second, in addition to CAPE in poplar propolis and Art-C in Brazilian green propolis, it is necessary to explore new bioactive substances in propolis. For example, besides CAPE, propolis are also very rich in other polyphenols such as p-coumaric acid and pinocembrin (22). It is therefore essential to investigate whether the other phenolic compounds in propolis also exert the same physiological activity. In addition, it may be a new breakthrough to study the combined effects of propolis polyphenolic compounds and other sources of phenolics. For example, Nishikawa’s team found that the combination of Art-C and curcumin induced the formation of beige adipocytes more strongly than Art-C or curcumin alone (86).

Finally, current research on the therapeutic effect of polyphenols in propolis on obesity is limited to animals and cell models. Therefore, clinical trials of the anti-obese effect of polyphenols in propolis are necessary.

## 5. Conclusion

Propolis is a resinous substance that bees collect to build and adapt their nests. Propolis is gaining popularity as a health supplement and is used in fields such as food, beverages, and folk medicine to improve health and prevent diseases such as obesity, diabetes, inflammation, and cancer. Propolis polyphenols are the key active components in propolis. In most cases, propolis polyphenols vary by different origins and plant sources, resulting in different pharmacological activities of these different types of propolis. Propolis polyphenols have superior weight loss activity. Currently, CAPE in poplar propolis and Art-C in Brazilian green propolis are the most widely studied anti-obesity components in propolis. Together, the anti-obesity mechanism of propolis and its derivatives mainly involving the following mechanisms, (I) reducing lipid accumulation and promoting lipid metabolism; (II) inhibiting adipogenesis by hindering adipocyte cycle progression and affecting the expression of adipokines; (III) inducing the transformation of white adipocytes into brown/beige fat cells, and promoting adipocyte thermogenesis by influencing creatine related metabolism and UCP1 dependent thermogenesis; (IV) regulating the structure and metabolism of gut microbes. In addition, PPAR γ plays a crucial role in the anti-obesity effect of polyphenols in propolis.

## Author contributions

JZ, HY, and GL: conceptualization. LZ: writing—original draft preparation. BZ, HL, and ZD: writing—review and editing. All authors contributed to the article and approved the submitted version.

## Funding

This work was financially supported by the Nanchang Science and Technology Talent Program Hongke Zi [2021] no. 185.

## Conflict of interest

JZ, HY, and GL were employed by Nanchang Concentric Purple Nest Biological Engineering Co., Ltd.

The remaining authors declare that the research was conducted in the absence of any commercial or financial relationships that could be construed as a potential conflict of interest.

## Publisher’s note

All claims expressed in this article are solely those of the authors and do not necessarily represent those of their affiliated organizations, or those of the publisher, the editors and the reviewers. Any product that may be evaluated in this article, or claim that may be made by its manufacturer, is not guaranteed or endorsed by the publisher.
